# A Drive-through Simulation Tool for Mass Vaccination during COVID-19 Pandemic

**DOI:** 10.3390/healthcare8040469

**Published:** 2020-11-09

**Authors:** Ali Asgary, Mahdi M. Najafabadi, Richard Karsseboom, Jianhong Wu

**Affiliations:** 1 Disaster & Emergency Management, School of Administrative Studies and Advanced Disaster, Emergency and Rapid Response Simulation (ADERSIM), York University, Toronto, ON M3J 1P3, Canada; 2 Advanced Disaster, Emergency and Rapid Response Simulation (ADERSIM), York University, Toronto, ON M3J 1P3, Canada; mirmahdi@yorku.ca; 3 Duty Officer, Departmental Emergency Operations Centre, Community and Health Services, The Regional Municipality of York, Newmarket, ON L3Y 4W5, Canada; Richard.Karsseboom@york.ca; 4 Department of Mathematics and Statistics and Laboratory for Industrial and Applied Mathematics, York University, Toronto, ON M3J 1P3, Canada; wujh@yorku.ca

**Keywords:** mass vaccination, point of dispensing, drive-through, mass immunization, COVID-19, agent-based simulation, discrete event simulation

## Abstract

Several research and development teams around the world are working towards COVID-19 vaccines. As vaccines are expected to be developed and produced, preparedness and planning for mass vaccination and immunization will become an important aspect of the pandemic management. Mass vaccination has been used by public health agencies in the past and is being proposed as a viable option for COVID-19 immunization. To be able to rapidly and safely immunize a large number of people against SARS-CoV-2, different mass vaccination options are available. Drive-through facilities have been successfully used in the past for immunization against other diseases and for testing during COVID-19. In this paper we introduce a drive-through vaccination simulation tool that can be used to enhance the planning, design, operation, and feasibility and effectiveness assessment of such facilities. The simulation tool is a hybrid model that integrates discrete event and agent-based modeling techniques. The simulation outputs visually and numerically show the average processing and waiting times and the number of cars and people that can be served (throughput values) under different numbers of staff, service lanes, screening, registration, immunization, and recovery times.

## 1. Introduction

The lockdown forced by the COVID-19 pandemic has reinforced efforts to find effective solutions to counter SARS-CoV-2. Although there are still lots of unknowns about how SARS-CoV-2 may evolve in the future, scientists believe that an effective vaccine, if invented and implemented globally, may be the best solution to end the COVID-19 pandemic [1]. Several institutions and laboratories around the world are working and sponsoring vaccine research and development to potentially help susceptible populations to become immune to contamination. According to the New York Times [2], from over 165 coronavirus vaccines that researchers are working on around the world, about 135 are in their preclinical stage (created in labs but not yet tested on human trials), 21 have entered Phase I (to be tested for safety and dosage on human trials), 13 are in Phase II (to be tested on an expanded number of human trials), 8 are in Phase III (to be tested on large-scale human trials), and 2 have already received approval for limited use. Some experts predict that an approved vaccination solution for large-scale implementation could be ready as soon as January 2021 or even earlier [1].

To bring the COVID-19 pandemic under control and substantially reduce hospitalization, morbidity, and mortality rates, and in the meantime reopen the economy, a large portion of susceptible people should receive the vaccine to become immune to the virus in a short period of time. Thus, similar to other deadly pandemic cases, as soon as vaccine production reaches the level that it can be offered to the general public, rapid mass vaccination should be implemented to minimize further human and economic impacts [1,3,4,5]. Such a large-scale implementation of the COVID-19 vaccine could be among the most challenging public health actions of the decade. From a preparation and planning point of view, this translates into many local mass vaccination sites in each city and town that offer immunization services.

However, vaccine development is only the first phase in the COVID-19 immunization process. After the successful development of COVID-19 vaccine, we will shift into vaccine production, distribution, and dispensing phases each with their own socio-economic and logistical challenges. Concurrent supply, distribution and dispensing of the vaccine to billions of people requires significant planning and preparation in all aspects of COVID-19 vaccination at international, national, and local levels. Since the ultimate goal of the vaccine process is to immunize the population against SARS-CoV-2, the success of vaccine development, production, and distribution very much depends on timely and efficient dispensing which requires extraordinary advance planning and preparation at different levels [6]. This includes, but is not limited to, vaccination prioritization, vaccination delivery methods, public awareness, and design of immunization and points of dispensing (PODs) facilities. In this context, the development of simulation tools that build capacities and enable such planning and preparation become very essential. 

One of the mass vaccination methods proposed in the literature and used in the past and during the COVID-19 pandemic for testing is the drive-through method. Drive-through clinics are particularly important in cases of communicable diseases because people wait in their own vehicles which limits virus transmission compared to walk-in clinics [7]. Studies and available experiences confirm that drive-through mass vaccination clinics offer many advantages and can be an efficient system for rapid and safe vaccinations [5,8,9]. However, rapid mass vaccination using the drive-through method requires thoughtful and comprehensive planning, design, trained human resources, and sufficient preparedness to enhance their effectiveness and efficiency [10].

This paper introduces a simulation tool developed for the design and operation of drive-through mass vaccination facilities. The tool is developed using a hybrid approach and by integrating discrete event and agent-based modeling methods. The simulation tool enables users to estimate how many people may be vaccinated and how many staff are needed to run such facilities efficiently under different setups and configurations. The simulation can help public health planners and decision makers to evaluate and understand the repercussions of their mass vaccination plans using drive-through options (e.g., different number of lanes, different number of personnel, different processing times that represent different processes). The rest of this paper is organized as follows: In Section 2, we present the current literature on mass vaccination and specifically on drive-through mass vaccination. In Section 3, we explain the modeling process and different components of the simulation tool. In Section 4, we demonstrate some of the model results. This is followed by a discussion and conclusion in Section 5, which also concludes the paper.

## 2. Background 

### 2.1. Drive-through in Public Health and Mass Vaccination

Mass vaccination has been a common strategy in controlling infectious diseases. In the case of COVID-19, once a vaccine becomes available for the general public or demand for mass flu vaccination becomes necessary, different mass vaccination methods can be used to ensure wider access to all segments of the society. Mass vaccination of COVID-19 will not only need to use the existing and traditional points of dispensing facilities such as clinics, pharmacies, schools, workplaces, nursing homes, pharmacies, and places of worships, but also requires the creation of new and innovative vaccination approaches. This is mainly because the use of some of the above listed facilities and settings may be inefficient or unsafe for immunization during a deadly infectious disease. While many of these settings have been used for mass influenza vaccination in the past, additional measures need to be considered when utilizing them for COVID-19 vaccination to ensure that vaccination sites maintain safe physical distancing [11].

Drive-through service delivery is not a new phenomenon. It emerged in the early 20th century with the massive use of automobiles in urban areas and continued to grow in different forms and shapes since then. The primary rationale for drive-through facilities is to provide more efficient services. While drive-through service delivery has a history of several decades now, its use in health services, particularly for mass vaccination purposes, has been very limited and only dates back to the late 1990s and early 2000s when health agencies started to add drive-through sites as one of their flu shot dispensing strategies [12,13,14].

In drive-through facilities, people are asked to drive to these facilities, register and sign the consent forms, receive the vaccine, and wait for a few minutes after that while staying in their vehicles for the entire time [12]. Previous studies have examined some of the direct and indirect advantages and disadvantages of drive-through facilities for mass immunization [5,7,14,15,16]. Some of the main advantages of drive-through facilities are: (1) low disease transmission risk for staff and public; (2) low exposure to virus (compared to closed settings); (3) large throughput; (4) reduced contamination of health-settings; (5) visitors’ comfort and protection; (6) serving people with mobility issues; (7) accessible to individuals in self isolation; and (8) useful for geographically scattered populations [17]. Some of the key limitations of drive-through facilities include: (1) weather conditions; (2) need for significant logistical preparation; (3) need large and suitable space; (4) clients that might faint in cars; (5) traffic issues; (6) difficulty in communication with people in their cars; (7) carbon monoxide exposure of staff; and 8) accessibility issues for those without cars [9]. 

The widespread research and use of the drive-through method for vaccination started during the 2009 H1N1 influenza pandemic (e.g., the work performed by Shim et al. [18]). Weiss et al. [7] assessed the feasibility of the drive-through mass vaccination method by analyzing a full-scale human simulation exercise for influenza vaccination in the USA. They found that the total median length of stay in a drive-through was 26 min. They did not find any significant carboxyhemoglobin increase in participants tested. They concluded that drive-through is a feasible and effective alternative to traditional walk-in clinics by providing faster vaccination under lower disease transmission risks. Reid [8] compared the efficiency of mass drive-through vaccination facilities versus walk-in clinics that were established in the US during the 2009 H1N1 influenza pandemic. The study examined several drive-through vaccination points of dispensing implemented by the Stanwood Camano Fire Department and some walk-in clinics implemented by local hospitals. The findings of this study indicated that drive-through facilities outperformed other mass vaccination facilities. In particular, the study found that the drive-through clinics were able to vaccinate more people, in a shorter time, while keeping the infectious risks lower.

Drive-through facilities have been frequently used in some countries during the COVID-19 pandemic for a wide range of health services including COVID-19 testing [19,20]. For example, Turrentine et al. [21] reported a drive-through that was set up for prenatal services during the COVID-19 pandemic in the US. In this case, the drive-through was used to protect health care providers and patients while providing access to this vital health service during the pandemic. They reported that these drive-through visits provide services that were not possible through telehealth and remote examinations, while reducing the need for clinical visits and thus reducing the anxiety of patients who prefer face-to-face visits. Drive-through facilities have also been expanded and used for providing pharmacy services during COVID-19 [22]. 

Another study also found that drive-through vaccination is mostly preferred to other options especially by those unwilling to leave their work to receive the vaccine. As reported by Bailey et al. [23], using a drive-through facility for seasonal flu vaccination for health workers as they entered their work places enabled health agencies to vaccinate more health workers in a shorter time frame. Bailey and her colleagues found that by using a drive-through facility, more health workers were vaccinated per 100 min during the 2018–2019 flu season conveniently and safely. The study highly recommends the use of this strategy for future pandemics. According to Nicola et al. [24] and Flynn et al. [16], due to the lack of enough traditional airborne infection isolation rooms, drive-through testing facilities are preferred for their shorter testing time, reducing infection among health workers and visitors by minimizing their exposure, reducing the costs of personal protection measures, and improving the efficiency and convenience for users.

Garrison [25] reported a mass drive-through testing facilities created in a large parking lot of a congregational Church in Birmingham, Alabama, US and reported that in this facility a total of 2216 people were tested over a five day period (17 to 21 March 2020). While 70 positive (3.15% overall screening yield) cases were reported among those tested in the facility, no cases of direct patient exposure or health worker contamination were reported. Similarly, Ton et al. [26] reported a drive-through testing site for COVID-19 that was created on the Mayo Clinic Florida campus and found that this was an effective method in minimizing patient contact and conserving personal protection equipment (PPE). Operating six hours per day, seven days a week, and consisting of three stations (patient identification and handout, assessment, nasal swab collection) staffed by two healthcare providers, this facility tested 1153 individuals. They also reported that drive-through can be considered as an effective testing strategy that conserves PPE during the COVID-19 pandemic.

Finally, Lee and Lee [27] studied drive-through facilities in South Korea for COVID-19 testing. Drive-through testing was first introduced on February 23rd as a low-cost, fast, and less risky method in the country. The design of drive-through testing facilities included several stations including registration, screening, swab sampling, and disinfection using a national standard procedure developed for drive-through testing facilities. Each test took an average of 10 min per person, which was much faster compared to alternative options that reported an average of 30 min. 

### 2.2. Design and Operational Challenges of Drive-through Facilities for Mass Vaccination 

Despite their advantages and promising outcomes, the planning, design, and operation of drive-through facilities for mass vaccination can be challenging [28] due to a number of factors related to the service area population, existing infrastructure, available logistics, communication, and coordination and partnership with different stakeholders such as first responders and humanitarian agencies [29,30,31]. The number and configuration of drive-through mass vaccination clinics should be planned based on the demographic attributes of the population, availability of adequate and accessible space and various staff to run the sites, traffic conditions, and vaccine supply and other logistical issues such as PPE. The number of sites in each community also depends on the size and spatial distribution of the population in service areas, availability of alternative mass vaccination clinics, percentage of the population able to use drive-through facilities, and targeted time frame for mass vaccination.

The scale of drive-through facilities depends on the availability and size of the available spaces, operational scope, available staff, and selected layout [12,28]. According to Lee et al. [32], space is a very dynamic requirement and different sites and scales can be used for drive-through mass vaccination facilities. When it is not be possible to create a large-enough drive-through facility to vaccinate the whole population [30], a number of smaller facilities can be set up. This can help in addressing some of the accessibility and traffic issues of drive-through facilities arising from a large number of cars trying to enter the drive-through clinic [5]. For example, a large drive-through vaccination facility in Ohio led to heavy traffic in the neighborhood [33]. To reduce traffic issues, drive-through vaccination clinics should: (1) be located near major roads, highways, or freeways; (2) have large access and exit points to support multiple lanes; (3) be large enough to accommodate multiple lanes for dispensing [33]; and (4) have adequate traffic control and safety plans to prevent traffic overflow onto adjacent transportation areas. 

Finding suitable locations for drive-through mass-vaccination facilities can also be a challenge. Drive-through clinics can be located in freeways, arterial highways, large parking lots, inside toll booths, in large sport fields, and on fairgrounds [34]. A drive-through site needs sufficient space to accommodate several vaccination lanes and their stations, a command post, an employee rest area, logistic and vaccination supply storage, IT and administration spaces, and safety and security vehicles and personnel [34]. For example, a large drive-through facility with 10 lanes is expected to have around 70 medical and non-medical staff per shift [28]. While suitable locations could be in private, public or government-owned spaces, public and government-owned properties are preferred due to logistic reasons and security precautions [34].

Various designs and layouts for drive-through vaccination sites have been suggested and used in the past [7,27,35,36]. A flu shot drive-through center usually has between two to ten lanes, three to four stations per lane that include triage and screening, registration, shot dispensing, and recovery and discharge. Drive-through vaccination sites with more dispensing lanes provide higher throughputs and can perform better in preventing overflow of traffic onto neighboring streets [28]. Overall, the layout of drive-through mass vaccination clinics can significantly affect their staffing requirements and overall efficiency. Lee et al. [32] reported that a design improvement increased throughput by 10%, increased the utilization of the facility by 18%, and decreased the waiting times in queues and vaccination stations between 10% to 85%. This facility served between 2000 to 3000 vehicles and their passengers in a day. 

Human resources management at drive-through facilities is another key issue. Each drive-through station requires an adequate number of medical and non-medical staff with different sets of skills. In addition to these, drive-through facilities are also supported and managed by incident command posts, requiring separate staff, space, parking lots, and security precautions. Moreover, large drive-through sites need to be maintained by local law enforcement, fire departments, emergency services, information technology service provider(s), and communication units of the responsible jurisdiction [23]. In terms of operation hours and number of shifts, a drive-through facility can operate 24 hours a day in three or four shifts depending on staff working hours, clients’ incoming rates in different times of the day, weather conditions, and lighting. 

Finally, attention must be paid to staff and visitors’ safety and security regarding road traffic, aggressive behaviors, extreme weather conditions, and virus transmission. Despite its low risk, infection prevention should still be a priority at drive-through facilities. Considering the transmissibility of the COVID-19 virus, proper protection of drive-through staff against the infectious disease is very important. Similarly, even though studies show that security and law enforcement agencies believe that controlling traffic at a drive-through clinic is an easily manageable task [37], it is important to implement all necessary risk reduction and safety measures. Furthermore, depending on the local weather conditions and location, the design of drive-through vaccination facilities should consider weather conditions such as temperature, humidity, wind, and rain. Although passengers receiving immunization are mostly protected in their vehicles against weather conditions, staff in drive-through vaccination sites may be less protected. In harsh weather conditions, a McDonald’s model of drive-through may be an alternative design option, in which immunization staff will reside inside a covered space or building, providing the immunization through an open door or window [31]. Effective use of drive-through facilities also requires public awareness campaigns to inform drive-through clients about what is expected from them at the clinic. Prior registration and appropriate clothing for vaccination purposes can save registration and vaccination times [38]. Finally, wearing a face mask reduces the risk of disease transmission to vaccination staff. 

### 2.3. Drive-through Mass Vaccination Modeling and Simulation Tools

Several mathematical models, agent-based, discrete event simulations, and multicriteria decision support systems have been developed and used for mass vaccination research and practice during the past two decades [5,17,34,36,39,40,41,42,43]. The main focus of these models and simulation tools are site selection, layout optimization, and resource and staff allocation [44,45]. Aaby et al. [36] developed an excel model named Clinic Planning Model Generator which helps decision makers to choose the size, number of patients vaccinated, and number of staff needed. They used data from a smallpox mass vaccination exercise conducted in Maryland (US) to calibrate their model and applied it to an influenza pandemic scenario. Washington et al. [46] developed a discrete event simulation tool to evaluate the capabilities and cost of mass influenza immunization clinics [5]. Wiggers et al. [9] and Gupta et al. [5] studied and simulated a drive-through mass vaccination facility that was set up in the city of Louisville during November 2009 as part of the HIN1 pandemic response. The facility was very large with 10 lanes. Using the Tecnomatix Plant Simulation software they developed their model based on data collected from an H1N1 drive-through vaccination clinic created in Louisville, Kentucky in 2009. They further extended this model to be applicable to large and small cities. They developed a customizable optimization tool for the design and operational planning of drive-through vaccination facilities for decision makers to determine the length and number of drive-through lanes, number of staff, and the resulting average waiting time. After providing model inputs including population that needs to be vaccinated, client arrival rates, processing times, and number of dispensing units, the model determines the required number of lanes, number and length of entrance lanes, number of lanes for handing out and filling in consent forms, number of detour lanes, personnel needed at different stops, and the average waiting time. 

Beeler et al. [37] worked on a discrete event simulation of a mass immunization clinic to improve human resources management of mass influenza immunization clinics. They developed their simulation using data from some Canadian clinics that responded to the H1N1 pandemic. The key attribute of this simulation is the inclusion of flu transmission risk in the simulation. They also showed that the marginal benefit of additional staff in mass vaccination facilities is underestimated when waiting costs and infection transmission are not taken into consideration.

Using a genetic algorithm approach, Ramirez-Nafarrate et al. [42] developed a mathematical model to optimize site selection and capacity of point of dispensing (POD) clinics. Their model allows decision makers to choose from potential mass vaccination points and suggests how to staff them to minimize average travel and waiting times.

Yaylali et al. [10] used simulation and optimization methods to assess and improve mass vaccination clinics. Using a combination of secondary and primary data, they developed a number of simulation tools for local mass vaccination clinics including drive-through and school-based facilities. They utilized discrete event models with 3D visualization of vaccination clinics using the Simio software. The model generates diverse mass vaccination clinic configurations that can be used to inform the design and configuration of future mass vaccination clinics. Authors claim that the 3D model of the clinics helps decision makers to better understand the mathematical models behind it, and to better compare various clinic setups. Finally, Glass [47] developed a discrete event simulation that provides the most optimal allocation of resources for mass vaccination clinics while minimizing medication errors.

A few mass vaccination site planning tools have emerged as the results of some of this research, including Maxi-Vac and RealOpt. Maxi-Vac was developed by the US Centers for Disease Control and Prevention in early 2000s in response to the growing threat of bioterrorism and potential need for mass vaccination in response. Maxi-Vac is an analytical tool that helps with the optimal design and staff setup of mass vaccination clinics that can have a range of one to nine stations. It shows how many people can be vaccinated under different arrangements of the clinic [46,48]. While this tool can be used for planning drive-through facilities, this type of planning is not its prime focus. 

Lee et al. (2009a) developed a decision support tool called RealOpt using a combination of mathematical modeling, simulation, and optimization engines, and coupled them with automatic graph-drawing tools and a user-friendly interface. RealOpt is a decision support tool that helps public health agencies to find suitable locations for mass vaccination facilities, to design preferred layout and determine staff resources need and their allocation, to perform disease propagation, analysis and monitoring, and to develop a variety of “what if” scenarios [5,17,32,34]. 

Despite the growing research and development in this area, there are still gaps in the current models that need further research. For example, according to Chiquoine [45], most of the existing models do not consider the impacts that arrival patterns have on model results, mass vaccination sites operations, and their transportation-related issues. Additionally, with the recent and continuous improvements in computer hardware and software, development of new models and simulations are becoming more possible and accessible. Finally, new diseases such as COVID-19 pose new challenges which require adjustments in the existing models and simulations.

## 3. Materials and Methods 

For the purpose of this research, we used the AnyLogic (version 8.6-AnyLogic North America, Oakbrook Terrace, IL, USA) simulation software. Our simulation tool is a combination of agent-based and discrete events modeling. The model contains a physical layout, several agents that interact with each other based on their predefined logic, and a model logic that implements the policies given to the model as user inputs.

### 3.1. Drive-through Layout

**Figure 1:**
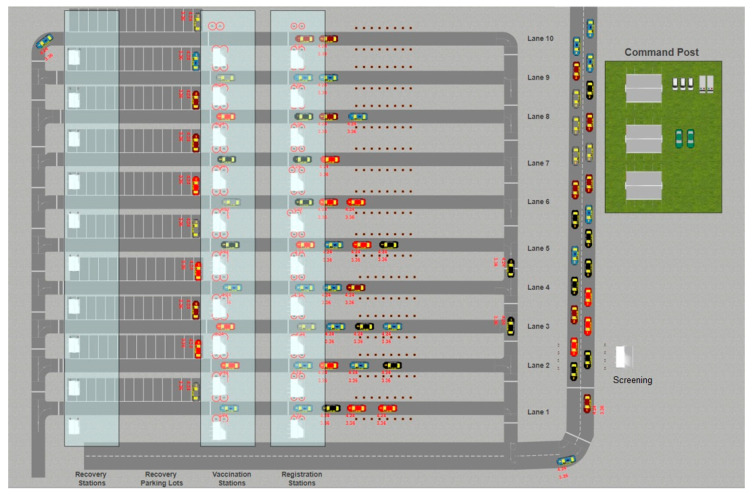
The 2D layout of the drive-through mass vaccination simulation tool.

The full layout (Figure 1) consists of ten service lanes, which can be turned on and off by the user at the beginning of the simulation. The area (except the command post side) is 150 m by 130 m. Cars enter the model at a given rate and go through a screening booth (a single service station before dispatching the cars into different lanes). A fraction of cars will be rejected at this stage and take the bypass extension to skip all service lanes and go out of the model. The rest of the cars will be disseminated to service lanes based on the existing queues in service lanes and based on user choices on High Occupancy Vehicles (HOVs) and Low Occupancy Vehicles (LOVs). Each lane consists of multiple booths (service stations) that offer registration (and consent to receive vaccination), delivering of the vaccination, and recovery. 

### 3.2. Drive-through Model Agents and Processes

Following modeling best practices, we have defined two super-agent types for agents with similar behaviors in different parts of the model. One super-agent pertains to the staff type (human agent) and the other is for different types of service stations. There are four subclasses in the model inherited from each of the super-agents above. 

**ABS_Staff** is a super-agent that represents all staff in the model. Each staff has several properties including parameters that connect it to PointNodes in the physical layout, variables that allow the staff to know the car being served at the time, as well as resource units that it will utilize at runtime. **Staff_S** represents the staff working in the screening booth. Since the model has only one screening booth and each booth has up to four staff, there can be up to four instances of this agent type in the model (i.e., the population of this agent will contain up to four agents). **Staff_R** represents the staff working in the registration booths. The model supports up to ten registration booths (one per service lane) and each booth has up to four staff. Thus, the population of this agent will contain up to 40 agents. **Staff_V** represents the staff working in the vaccination delivery booths. The model supports up to ten vaccination booths (one per service lane) and each booth has up to four staff. Thus, the population of this agent will contain up to 40 agents. **Staff_RE** represents the staff working in the recovery booths. The model supports up to ten recovery booths (one per service lane) and each booth has up to four staff. Thus, the population of this agent will contain up to 40 agents.

The specific service stations instantiated from the service super-agent are: a screening booth; a number of registration booths; vaccination booths; and recovery booths. There is only one screening booth in the model, but since the model supports up to ten service lanes, there can be up to ten instances of each type of the rest of the service stations, as described here.

**ABS_Station** is a super-agent that represents service stations in the model. Each station has several properties including parameters that connect it to PointNodes in the physical layout, variables that allow the station to know the car being served at the time, as well as resource units that it will utilize at the runtime. Four subagent types are created from this abstract agent type, as follows: (1) **St_Screen** represents screening station, and the model has only one instance of it. This agent contains the population (instances) of the Staff_S agents (up to four, as stated above); (2) **St_Register** represents registration stations. The model populates one registration station instance per active service lane. Thus, there can be up to ten registration service stations in the model. The St_Register agent type contains a statechart that keeps track of the state of its instances, and toggles between the “idle” and “serving” modes accordingly. This agent also contains the population (instances) of the Staff_R agents (up to four, as stated above, per station); (3) **St_Vaccine** represents vaccination delivery stations. The model populates one vaccination station instance per active service lane. Thus, there can be up to ten vaccination service stations in the model. The St_Vaccine agent type contains a statechart that keeps track of the state of its instances, and toggles between the “idle” and “serving” modes accordingly. This agent also contains the population (instances) of the Staff_V agents (up to four, as stated above, per station). **St_Recovery** represents recovery stations. The model populates one recovery station instance per active service lane. Thus, there can be up to ten recovery service stations in the model This agent contains the population (instances) of the Staff_RE agents (up to four per station).

**Figure 2:**
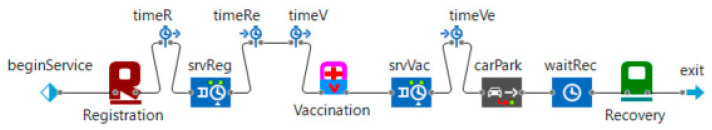
The service lanes flowchart of the drive-through mass vaccination simulation tool.

Besides the human and non-human agents listed above, there are other agents defined in the model, as described here. **ServiceLane** is a less tangible agent type. In fact, the redundant nature of service lanes calls for designing this agent type to allow replication of one logic for all service lanes. As explained before, each service lane contains its specific registration station, vaccination station, and recovery station. The logic of how an incoming car enters the service lane, moves from a station to the next station, and finally leaves the service lane, is defined by a flowchart in the ServiceLane agent type (Figure 2).

The number of service lanes created in the model depends on the user input and can vary between one and ten. **Car** agent represents incoming cars, entering the drive-through clinic for receiving vaccination services. The incoming cars are generated at a user-defined rate, and as soon as they leave the recovery area, they are deleted from the model. There are separate model variables that record the total count of cars that enter the clinic. The car agent type contains variables to keep a calculated delay time per service station based on a number of given variables, explained in the Model Logic. The car agent has a statechart through which the service station of the car is maintained. Each instance of the car contains a specific population of the Passenger agent.

**Passenger** agent is the most granular agent type in the model, which represents a car passenger. Each car can have between one and five passengers, and each passenger is either an adult or a child (non-adult). The difference between the two is in the registration phase, as the non-adult should give consent through one of his/her accompanying adults, which assumedly takes less registration time.

### 3.3. Simulation Inputs and Outputs 

**Figure 3:**
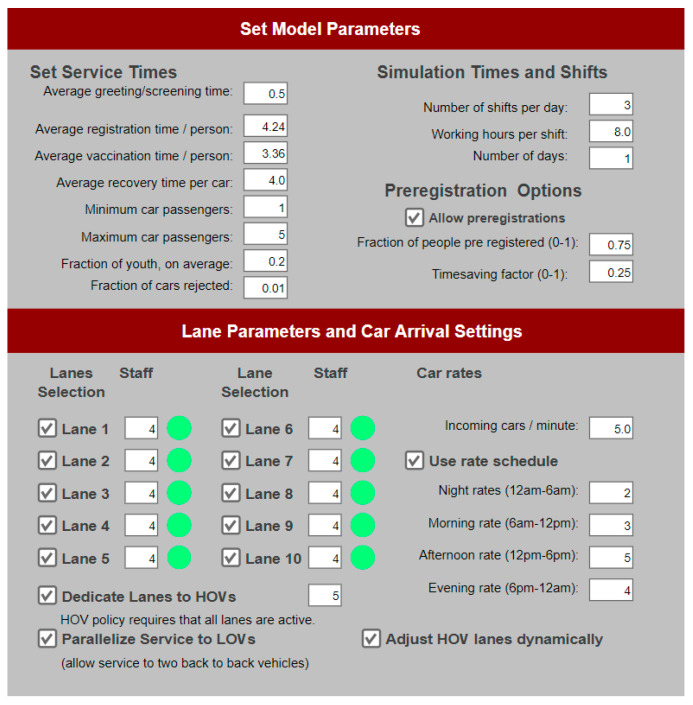
Simulation setup panel.

To allow user control over the simulation, several parameters are set by user input before the simulation run begins. Below is a list of all user inputs and their default values, as well as their acceptable range (if any). The user input panel is also shown in Figure 3.

- Open service lanes (minimum: 1; maximum: 10; default: 10).
- Number of staff serving in each open service lane (minimum: 2; maximum: 4; default: 4).
- Choice to dedicate lanes to High Occupancy Vehicles (HOVs) to allocate specific lanes to them or not. This policy requires and enforces that all ten service lanes be in operation.
- Number of lanes assigned to the HOVs (minimum: 1; maximum: 9; default: 5).
- Choice to automatically optimize the number of HOV lanes according to the last hour queue patterns.
- Choice to parallelize service where possible (if enough staff are available at the LOV station) to Low Occupancy Vehicles (LOVs). This policy choice is only available if the HOV choice is selected.
- Choice to allow pre-registration of clients (default: false), as well as the fraction of clients (at car level) that preregister (default if pre-registration is chosen: 75%), and the timesaving factor that becomes effective for the pre-registered clients (default if pre-registration is chosen: 25%);
- Average registration and vaccination times per person (default: 6.44 and 5.36 min respectively).
- Average recovery time per car (default: 5 min).
- Minimum and maximum passengers in a car (default: 1 and 5, respectively).
- Fraction of dependent children (except the driver) on average (default: 20%).
- Number of incoming cars per minute (default: 5).
- Fraction of cars rejected from the screening booth (default: 1%).
- Number of shifts per day.
- Working hours per shift.
- Number of days (for the whole simulation run).

**Figure 4:**
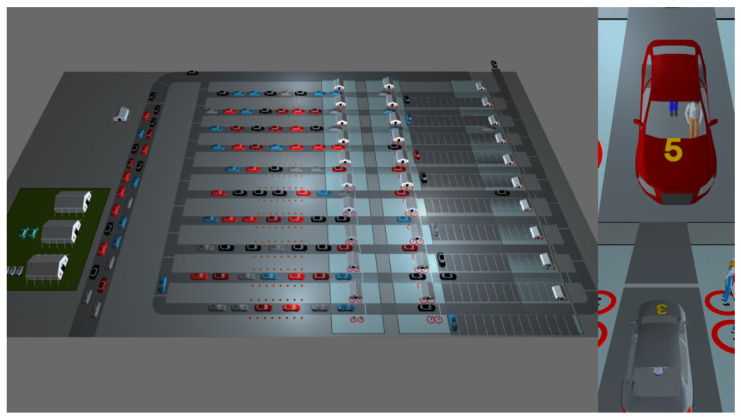
3D view of the drive-through mass vaccination simulation tool.

Model outputs can be viewed as 2D and 3D models (Figure 4), charts, and dashboard. 

## 4. Results

In this section we present the results of several experiments including parameter variation and sensitivity analysis of the proposed simulation model. The base experiment is one realization of the simulation. 

### 4.1. Base Experiment

We ran the simulation with all lanes open in full capacity (four staff in each station). This runs the model for 1 day with three shifts under a fixed rate of 5 incoming cars per minute. Parameter settings for this experiment are shown in Table 1. The user can change all the parameters from the user control panel before running the simulation.

**Figure 5:**
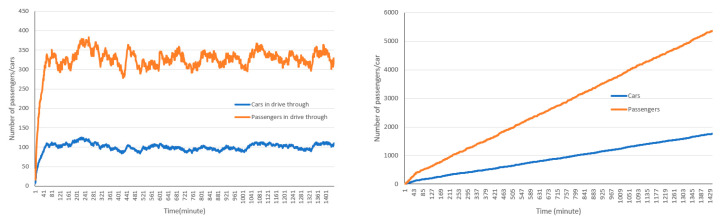
Cumulative and non-cumulative number of cars and passengers using the drive-through facility.

The cumulative and non-cumulative number of cars and passengers that entered and exited the drive-through are presented in Figure 5. In this model setting, a total of 1771 cars and 5330 passengers used the drive-through, while around 120 cars and around 290 to 380 passengers were in the drive-through after reaching its full capacity.

**Figure 6:**
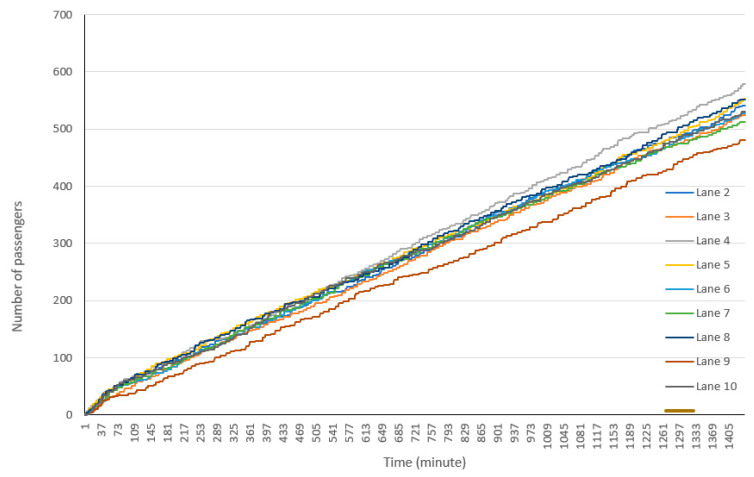
Number of passengers using the drive-through facility by lanes.

Figure 6 shows the total number of passengers served in each lane. Some differences are observed among the total number of cars and passengers for each lane.

**Figure 7:**
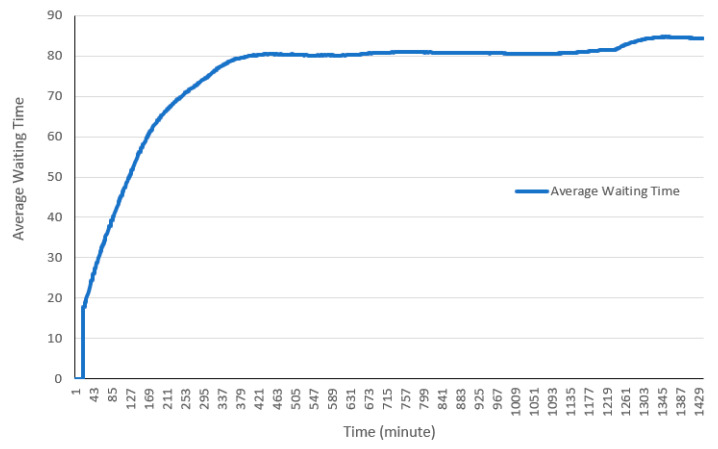
Average waiting time in the drive-through.

As illustrated in Figure 7, most cars spend between 80 to 90 min in the drive-through on average. Early users spend less time in the drive-through, however as more cars enter the drive-through and queues are formed, the wait time and thus the total time spent in the drive-through increases. The maximum time spent in the drive-through in this run is 96 min.

### 4.2. Parameter Variations and Sensitivity Analyses

In this section we present the simulation results by varying different parameters and options including number of lanes, number of staff in each station, and pre-registration.

#### 4.2.1. Number of Lanes

**Figure 8:**
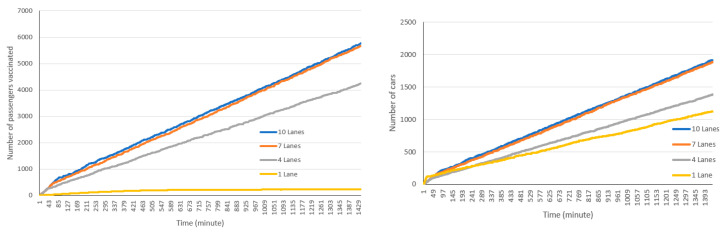

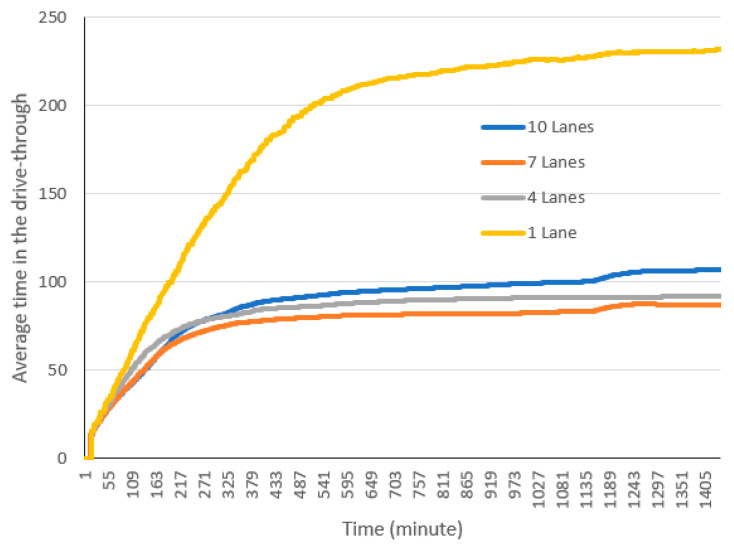
Number of passengers (**top left**) and cars (**top right**) and average time spent in the drive-through (**below**) using different numbers of lanes.

The simulation tool allows for running the drive-through with different lanes and configurations (all first five lanes, alternate lanes, etc.). Figure 8 presents the simulation results for different numbers of open lanes. In these experiments, we opened consecutive lanes starting from lane 1. As shown in Figure 8, as the number of open lanes increases, the total number of cars and passengers in the drive-through also increase. As expected, by opening more lanes the average time spent in the drive-through facility decreases. For example, when one lane is operating, the average time spent in the drive-through for each car is very high and reaches close to 180 min. However, when 10 lanes are opened, the average time spent in drive-through decreases to around 75 min.

#### 4.2.2. Number of Staff in Each Station

**Figure 9:**
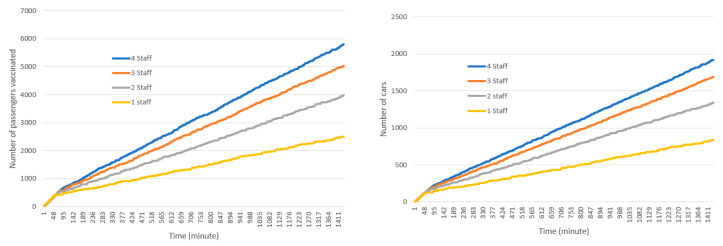

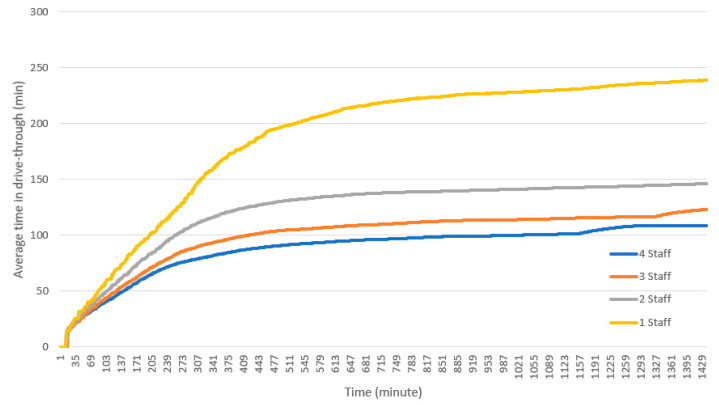
Simulation results (passengers, top left; cars, top right; average waiting time, bottom) under different numbers of staff in each station.

The simulation currently considers a minimum of one and a maximum of four staff in each station, however, the staff number can be adjusted to include more if needed. It is assumed that each staff provides service to one passenger at a time and thus additional staff can provide additional service if the incoming cars in the lanes have more than one passenger. In other words, if the number of staff in each station is four, all four staff can provide service concurrently in cases that incoming cars have four or more passengers. Because in this model, the incoming cars choose the lane with minimum cars in the queue, there are a considerable number of occasions where this situation can occur. Figure 9 shows the cumulative number of passengers, cars, and the average time spent in the drive-through under various numbers of staff in each station: 1, 2, 3, and 4 staff respectively. As illustrated in this figure, increasing the number of staff increases the number of cars using the drive-through and passengers receiving the service. Furthermore, as a result of this increase in staff, the average time spent per car in the drive-through decreases.

#### 4.2.3. Pre-Registration

**Figure 10:**
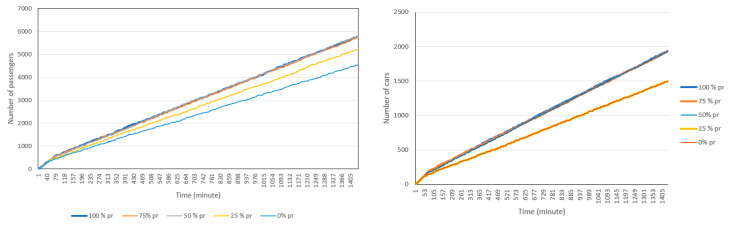

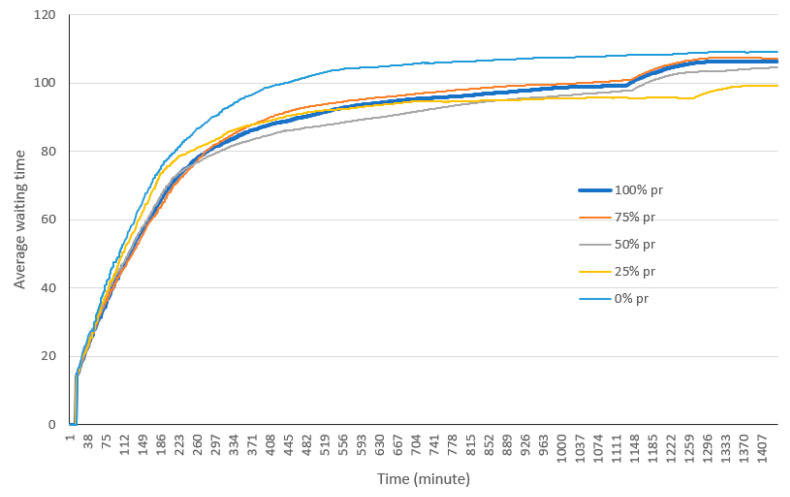
Total number of passengers and cars in drive-through when allowing for pre-registration and assuming that pre-registration reduces the registration time by 50%.

Pre-registration is one way to increase the efficiency of the drive-through vaccination by reducing the registration time, which is the lengthiest stage in the vaccination process. Pre-registration can be done through an online registration portal. In this experiment we allow the simulation to run with a pre-registration option but with different percentages of pre-registration. We assumed that 100%, 75%, 50%, 25%, and 0% of passengers have pre-registered for vaccination in respective simulation runs. By doing so, the registration time is reduced by 50%. Although not currently included in this simulation, it is possible to allocate special lanes to pre-registered visitors that significantly reduces their waiting times, to encourage more pre-registration. Figure 10 shows the cumulative (a) and non-cumulative (b) number of passengers and cars in the drive-through when allowing pre-registration. As expected, pre-registration yields higher throughput in terms of the total number of passengers using the service and decreases the average time spent on the drive-through in some cases (but not always).

#### 4.2.4. Arrival Rate and Schedule

**Figure 11:**
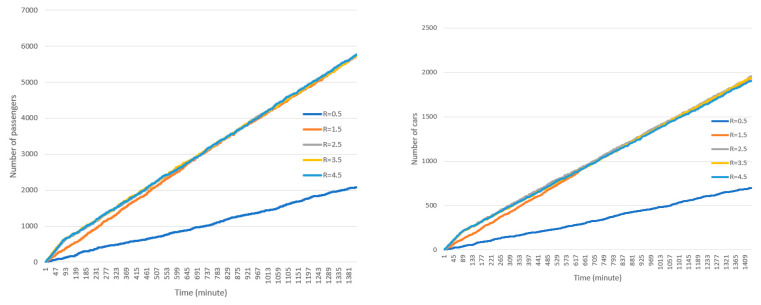

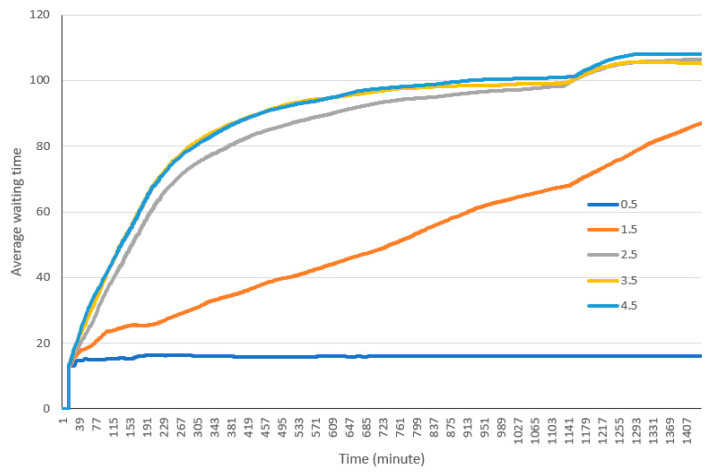
Number of passengers (**top**) and cars (**bottom**) and average time in drive-through (**right**) under different arrival rates.

Arrival rate can be defined in a number of ways including fixed rates, arrival schedule, arrival timetable, etc. In this experiment we run different experiments with different arrival rates ranging from 0.5 cars per minute to 5 cars per minute (Figure 11). The results show that an increase in the arrival rate increases the number of people receiving service and also increases the average time spent in the drive-through. However, these values remain unchanged with higher values of arrival rates due to the unchanged (and limited) capacity of the drive-through facility.

Because a fixed arrival rate, particularly when the drive-through is running for 24 h, may not be realistic, we ran an experiment in which car arrival rate changes during different hours of the day. 

**Figure 12:**
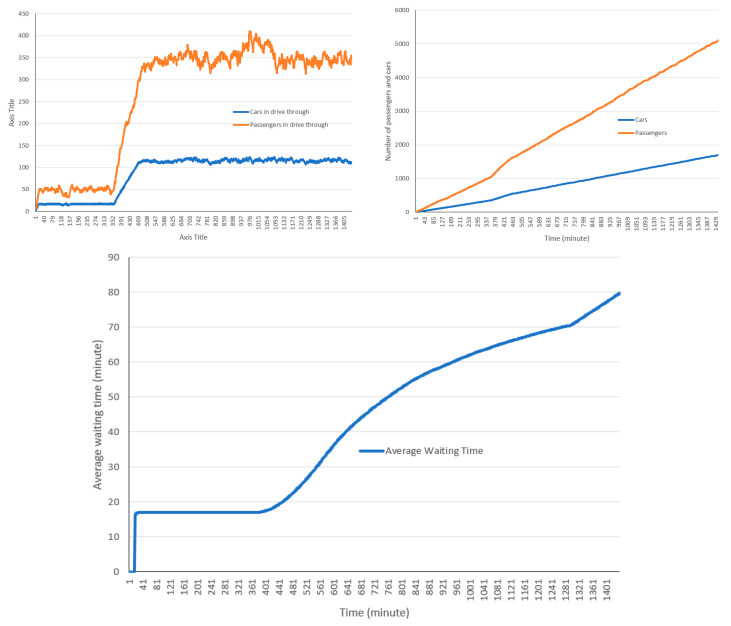
Non-cumulative and cumulative number of passengers and cars (top right and left panels) and average time in drive through using a hypothetical schedule rate (12 a.m. to 6 a.m. = 1; 6 a.m. to 12 p.m. = 2, 12 p.m. to 6 p.m. = 4, 6 p.m. to 12 a.m. = 3).

Figure 12 shows the number of passengers, cars and average time spent in the simulated drive-through under various arrival rates using the rate schedule option in the AnyLogic simulation software. In this example a lower arrival rate has been considered for the night hours (12 a.m.–6 a.m.) and higher values have been assigned to daytime (6 a.m.–12 p.m. and 12 p.m.–6 p.m.) and evening hours (6 p.m.–12 a.m.). The results show that arrival rates yield lower number of cars and passengers and significant variations of average processing time throughout the day.

We have made a version of this model available online on the AnyLogic Cloud platform, for everyone to experience the settings and view the results [49].

## 5. Discussion 

We introduced a simulation tool for drive-through mass vaccination clinics in anticipation of the need for mass vaccination of people when a vaccine becomes available for SARS-CoV-2. Such tools can help with the initial planning and design of mass vaccination facilities. We used AnyLogic simulation software to develop the model as it provides opportunities for more effective visualization (2D and 3D), scenario analysis, and optimization. Moreover, agent-based capabilities of the AnyLogic platform allow users to add agent-specific attributes and behaviors regarding immunization, virus transmission, and interactions between the service providers and service receivers. 

The results presented in the results section were generated by one realization of the simulation for demonstration purposes. The parameters here have been set using the screening, registration, vaccination, and recovery rates used in some of the previous cases of mass vaccination using drive-through facilities. While the results of our simulation are close to some of the previous studies with similar parameters and structure, some parameter values may be different in the case of the SARS-CoV-2 vaccine, depending on the differences that may exist in the registration, vaccination, and recovery protocols for this specific immunization, most of which are not available yet. 

Due to some stochasticity in the model, the results can be different in each run, however, the variations are minor. For example, we ran a Monte Carlo simulation with the same parameter settings with 100 iterations and found that the difference between the lower and upper bounds of our output variables were relatively small. For instance, the number of cars served ranged between 1890 to 1960 the number of passengers vaccinated ranged between 5740 to 5900, and the average vaccination processing time changed from 99 to 111 min. 

Since it is most likely that when the SARS-CoV-2 vaccine becomes available, health workers working in mass vaccination facilities will receive the vaccine beforehand, it is possible that the virus transmission concern for health workers is reduced. However, all protection measures and protocols still need to be strictly followed to prevent potential transmission from passengers to the health workers. 

This simulation model can provide some insights regarding different drive-through layout settings in terms of number of lanes, staff available at each lane, pre-registration, allocation of high occupancy vehicles, and parking arrangement. The results of this simulation correspond to the findings of previous models and simulations [17] which argue that appropriate staffing and the layout of the drive-through vaccination facilities are very important factors contributing to the optimal use of drive-through facilities. Our results show that under the examined layout, an increase in the number of lanes or staff reduces the average time spent in the drive through and increases the total number of passengers vaccinated in a nonlinear manner. For example, increasing the number of staff in each station does not necessarily increase the efficiency of the drive-through. This measure should be used in conjunction with other measures, particularly the ability to sort incoming vehicles based on the number of passengers in them. This issue is addressed in our simulation by the ability of the model to dynamically adjust the vehicle capacities to lanes, i.e., the user can check a box to let the model dynamically allocate enough HOV lanes based on the past hour averages of the HOV queues and non-HOV queues. The simulation model also allows parallelization of the service delivery to vehicles with one or two passengers in case there are enough staff available at the service booth. 

Public health agencies can use our simulation tool to examine how many people can be vaccinated for a given number of days, shifts, and working hours per shift. Moreover, the model can help decision makers to have an estimate of how many drive-through facilities would be needed to achieve a certain number of immunizations in a specific time period. These insights can also be used to decide on suitable locations of additional drive-through sites considering the space and traffic conditions of the potential sites.

According to previous studies, the registration stage contributes most to the formation of bottleneck in mass vaccination systems including drive-through facilities [45]. Suggestions have been made to reduce the registration time by providing registration forms and vaccine information to visitors in advance. Thus we added an option for the online pre-registration that allows for a certain amount of registration to be done beforehand. Pre-registration can be implemented through a secure online system that registers visitors and provides them with the information they need before coming to the drive-through for vaccination. Such a system can be enhanced further to allow users to even book their preferred vaccination time and date. Additional measures can be taken to prioritize the prescheduled visitors over others to encourage pre-registration.

Another important point is that the car arrival rates impacts the results significantly [40,41,42,45]. In the reported experiment in this paper (the base run), we used a fixed arrival rate of 5 cars per minute, but this arrival rate does not have to be fixed and can vary during the day depending on demographic and environmental factors, especially if no pre-registration arrangement has been taken into consideration. This arrival rate has significant impacts on the number of people being vaccinated and the average time spent in the drive-through facility as shown in Figure 11 and Figure 12. Therefore, depending on the drive-through configurations, it is important to implement strategies similar to pre-registration to better manage the incoming traffic.

The service times used in our model have come from previous studies based on non-COVID-19 mass vaccination drive-through facilities. If the service times for COVID-19 vaccination turn out to be different, we need to use these numbers in the simulation to allow a simulation run with the updated values. 

Finally, the model presented in this paper has some limitations that need to be addressed in future versions and as more information about SARS-CoV-2 becomes available: (1) This simulation uses only one geometrical layout to analyze different strategies. Studying strategies and different policy choices under different geometrical topologies can yield different results that require further study. (2) Another limitation of this simulation that demands further work is consideration for additional behavioral and user needs, such as the ability of the simulation to allow people who change their mind after they enter the vaccination line to drive out, people who need further recovery time and might even need to be taken care of outside their vehicles in a caregiving area, potential accidents between vehicles, users’ need for washrooms, etc. (3) In this model we have not considered disease transmission possibilities. However, the simulation can be expanded to account for SARS-CoV-2 contamination possibilities. This enhancement will enable us to understand potential disease transmission scenarios if infected passengers or health care providers exist in the system. This will also help in comparing the disease transmission implications of the drive-throughs with other mass vaccination clinic types. (4) In this simulation we have assumed the continuous and sufficient supply of vaccine to the drive through. The model can be expanded to include vaccine supply issues and their impacts on the model outputs. (5) In this simulation we did not focus on different transportation vehicle types such as bicycles, motorcycles, public transit, etc. Our assumption was that to prevent infection, large occupancy public transit vehicles and minibuses are not used for drive-through vaccination, however, one can allocate certain lines to bikes, motorcycles, and taxis. (6) It is also equally important to note that this simulation assumes the availability of vaccine for mass vaccination. Realization of this will depend on the vaccine production, distribution, and public health agencies’ immunization policies that were beyond the focus of this study.

## 6. Conclusions

In this paper, we introduced a hybrid simulation model that utilized agent-based and discrete-events methods to visualize a drive-through mass vaccination clinic. During the COVID-19 pandemic, drive-through mass vaccination clinics can be an optimal option for reducing contacts between healthcare workers and the people who are seeking to receive the vaccine treatment. This is particularly effective for communities where access to suitable sites and private cars are high. Drive-through have a high throughput and are easy to setup and operate. The model presented here has a public interface that is available online and can be used by the healthcare decisionmakers to plan for their actual drive-through mass vaccination clinics, by setting input variables and getting the results. The model can provide scenario-based outputs that can help decisionmakers to come-up with an optimal clinic design for their purpose.

## Figures and Tables

**Table 1:** Parameter values for the base experiment.

Parameters	Value
Greeting and screening time per car (minute)	Uniform (0.25, 0.5)
Average registration time (minute) per passenger	4.24 *
Average vaccination/dispensing time per passenger (minute)	3.36 *
Minimum number of passengers	1
Maximum number of passengers	5
Fraction of non-adult passengers	0.2
Number of incoming cars per minute	5
Fraction of cars rejected at the screening	0.01
Number of shifts per day	3
Hours of operations in each shift	8
Number of days	1
Lanes open	1, 2, 3, 4, 5, 6, 7, 8, 9, 10
Staff in each station	4
Pre-registration	No
High occupancy vehicle lane	No

*. Based on Wiggers et al. [9].
